# Phylogenetic Profiling: How Much Input Data Is Enough?

**DOI:** 10.1371/journal.pone.0114701

**Published:** 2015-02-13

**Authors:** Nives Škunca, Christophe Dessimoz

**Affiliations:** 1 ETH Zürich, Department of Computer Science, Universitätstr. 19, 8092 Zürich, Switzerland; 2 Swiss Institute of Bioinformatics, Universitätstr. 6, 8092 Zürich, Switzerland; 3 University College London, Gower St, London WC1E 6BT, UK; Laboratoire Arago, FRANCE

## Abstract

Phylogenetic profiling is a well-established approach for predicting gene function based on patterns of gene presence and absence across species. Much of the recent developments have focused on methodological improvements, but relatively little is known about the effect of input data size on the quality of predictions. In this work, we ask: how many genomes and functional annotations need to be considered for phylogenetic profiling to be effective? Phylogenetic profiling generally benefits from an increased amount of input data. However, by decomposing this improvement in predictive accuracy in terms of the contribution of additional genomes and of additional annotations, we observed diminishing returns in adding more than ∼100 genomes, whereas increasing the number of annotations remained strongly beneficial throughout. We also observed that maximising phylogenetic diversity within a clade of interest improves predictive accuracy, but the effect is small compared to changes in the number of genomes under comparison. Finally, we show that these findings are supported in light of the Open World Assumption, which posits that functional annotation databases are inherently incomplete. All the tools and data used in this work are available for reuse from http://lab.dessimoz.org/14_phylprof. Scripts used to analyse the data are available on request from the authors.

## Introduction

Phylogenetic profiling is a technique to infer gene function from patterns of presence and absence across species, using the principle of “guilt-by-association”: if homologs, genes that share ancestry, are inherited or lost co-dependently, they are likely to be functionally or physically interacting. In a now classic paper, Pellegrini *et al*. first described this method of analysing biological processes on a genome scale [1]. By tracking the patterns of presence or absence of *Escherichia coli* genes in different organisms, Pellegrini *et al*. showed evidence that proteins with similar patterns of presence or absence—similar phylogenetic profiles—tend to share functional annotations. The many extensions of this approach (e.g., [2–4]) provide further evidence of its usefulness.

Various studies looked at aspects of phylogenetic profiling such as the genomes included [4, 5], vocabulary used in functional annotation [6], methods used to find or group homologs [7–9], and methods used to find similar phylogenetic profiles [10] (see [11] for a review). For example, looking at the genomes included, one study suggests that reference genomes should be selected from moderately and highly genetically distant organisms, from all three domains of life [2]. Another study noted a drop-off in performance with increased number of Eukaryotes [4]. In addition, Jothi *et al*. note that the over-representation of parasitic Eukaryotes and vertebrates additionally make Eukaryotes less useful in reference sets. Another study showed that phylogenetic profiles (and some other genome context methods) substantially improve in performance when a subset of phylogenetically diverse Archaeal genomes were used with Eubacteria [3].

Regarding functions that can be predicted using phylogenetic profiles, those related to translation—a conserved cellular pathway—should have high predictive accuracy when the phylogenetic diversity of organisms included in the profiles is highest [4]. Jothi *et al*. [4] noted that performance is the worst for the translation system when they use only the superkingdom to which the test-organism belonged, e.g., when the test organism is *E. coli* and the superkingdom is Bacteria. In line with these results, in a recent benchmark study of genome-context methods based on KEGG pathways, Muley and Ranjan [3] showed that phylogenetic profiling performs well for genetic information processing pathways (“translation,” “folding, sorting, and degradation,” and “replication and repair”). Pathways involved in motility were best predicted by phylogenetic profiling. Indeed, since these pathways are restricted to motile organisms, they show a strong co-occurrence pattern.

Phylogenetic profiling has been successfully used to discover protein-protein interactions and to annotate new genomes, particularly in prokaryotes [11]. These successes can be attributed to a combination of methodological refinements [7, 12–14], increase in the number of sequenced genomes, and increase in the number of functional annotations—Gene Ontology (GO) annotations in particular [15]. However, whereas most of the previous literature on phylogenetic profiling has focused on methodological refinements, comparatively little is known about the relative contribution of more genomes and more functional annotations. As there are now about 17,500 bacterial genomes sequenced [16], including all of them is not practical. Wanting to use phylogenetic profiling to functionally annotate a new bacterial genome, a researcher might thus ask: how much input data is enough to achieve good functional predictions?

Here, we sought to answer this question by decomposing the effect of additional genomes and additional functional annotations on phylogenetic profiling predictive accuracy. In our tests, phylogenetic profiling generally improved with more input data, but the marginal benefit of additional genomes rapidly diminished beyond ∼100 genomes and practically vanished beyond ∼400 genomes.

In general, the availability of new annotations is greatly beneficial for phylogenetic profiling: the annotations added in the new releases of the UniProt-GOA database have drastically increased the predictive accuracy of phylogenetic profiling. Conversely, decreasing the number of annotations lead to a clear detrimental effect on the overall predictive accuracy. For a minority of relatively general GO terms, there are already sufficient annotations in the database to train accurate predictors of functional annotation, but for most GO terms, annotations are still lacking.

Previous work has purported the view that genomes need to be carefully selected, typically by maximising the phylogenetic diversity among the genomes [3–5]. Our analyses confirmed that this is the case and that, conversely, considering only particular subclades decreased predictive accuracy. We also observed that while maximising phylogenetic diversity lead to the best results, the simple strategy of randomly selecting subsets from all available genomes was nearly as effective.

Finally, we show that our results hold even when we account for the lack of comprehensive annotations in biological databases. Because we can never be sure whether particular gene products are comprehensively annotated—biological databases are subject to the Open World Assumption [17]—we introduce a new testing framework based on subsets of well-annotated proteins and sparsely-annotated ones, and ascertain that our conclusions hold under the different conditions.

## Results and Discussion

First, we show that increasing the amounts of data in biological databases has led to better predictive accuracy of phylogenetic profiling. Then, we separately consider the influence of the amount of annotations and the number of sequenced genomes. We also discuss the problem of selecting optimal subsets of genomes. Finally, we provide evidence that our conclusions hold in light of the Open World Assumption—the notion that functional annotation databases are incomplete [17, 18].

### Phylogenetic profiling strongly benefits from more data

To evaluate the influence of new annotations and newly sequenced genomes on phylogenetic profiling, we first retrieved a set of 1093 GO terms for which we can reliably assign annotations using the 2013 UniProt-GOA database (see Methods). We then retrospectively applied our phylogenetic profiling method on successive past versions of the UniProt-GOA database, comparing the predictive accuracy of the method on these sparser datasets.

**Figure 1:**
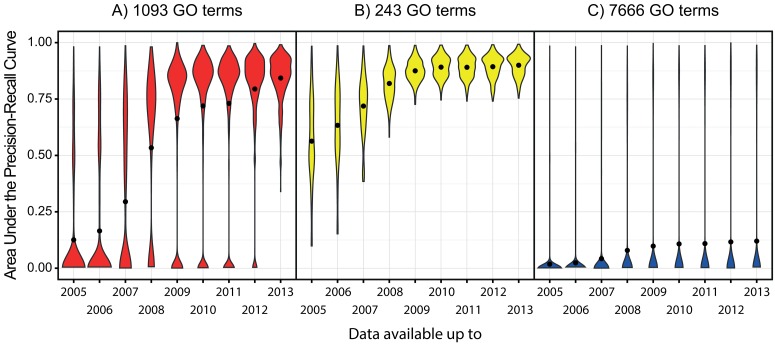
Predictive accuracy of phylogenetic profiling, measured in Area Under the Precision-Recall Curve (AUPRC), when we change the amount of data available for training the model for functional annotation. For each year from 2005 to 2013 denoted on the x-axis, the corresponding dataset includes those genomes that were available both in the OMA database, as well as the NCBI taxonomy database in the respective year; the phylogenetic profiles are annotated using the UniProt-GOA file available in January of the respective year. Each violin plot summarizes the distribution of GO terms according to the AUPRC value: the area of the plot corresponds to the probability density of GO terms at different values of AUPRC; the black dot denotes the mean value of AUPRC. A) We consider 1093 GO terms in total—those that had sufficient annotation information in the most recent database releases. If the model does not have enough data to infer annotations for one of the 1093 GO terms, as will be the case for, e.g., 846 of these GO terms using the data from 2005, its AUPRC score is zero. B) We consider only the GO terms that had sufficient annotation information throughout the analysed releases. C) We consider all the GO terms from the prokaryotic GO set.

For these GO terms, the combination of the newly sequenced genomes and the new annotations had a favourable effect: on average, the predictive accuracy of phylogenetic profiling steadily improved each year (Fig. 1A). The difference in predictive accuracy between the annotation model available in 2005 and 2013 is extreme.

For some GO terms, the earlier releases of the database do not have enough data to infer annotations: for example, the 2005 annotation database has insufficient information for 846 of the above 1093 GO terms; there, AUPRC is zero resulting in the heavy base of the first series in Fig. 1A. In the second set of experiments, we focused on the GO terms that had sufficient annotation information throughout the analysed releases, thereby evaluating the influence of new annotations and newly sequenced genomes on this smaller—and more general—set of 243 GO terms.

Just like in the first set of experiments, in the second set of experiments the average predictive accuracy increases with more data (Fig. 1B). However, here we see very little increase in predictive accuracy after 2010: it is tempting to speculate that for this set of GO terms we have approached the maximum predictive accuracy that our implementation of phylogenetic profiling can achieve, regardless of the number of newly sequenced genomes or new annotations.

In our third set of experiments, we supplemented our set of 1093 GO terms with all the GO terms that can be assigned to prokaryotic proteins, thereby obtaining 7666 GO terms. Even using the most recent release of the database, for 6573 (7666–1093) of these AUPRC will be zero, as we do not have enough data to make a prediction (Fig. 1C). Considering the data from this angle shows that the functional annotation of genomes remains largely incomplete: despite assigning many GO terms with high AUPRC values and seeing a general trend of increasing predictive accuracy, there are many more GO terms for which we cannot make predictions because of the lack of annotations.

In all three sets of experiments, the observed increase in predictive accuracy is a consequence of both the newly sequenced genomes, which increased the number of genomes included in the phylogenetic profiles, as well as the new functional annotations. In what follows, we separately evaluate the influence of each of these two factors, focusing on the most recent database releases available and the set of 1093 GO terms we could therewith reliably predict (see Methods).

### How many annotations are enough?

**Figure 2:**
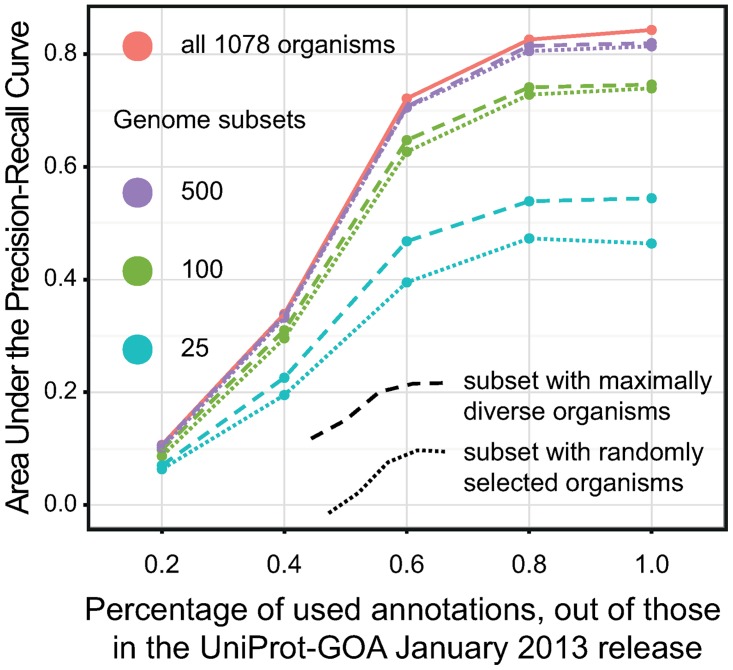
Predictive accuracy of phylogenetic profiling, measured in AUPRC, when we reduce the number of annotations used for phylogenetic profiling. For each of the experiments denoted on the x-axis, we only used a fraction of the available annotations in the most recent dataset. Dashed and full lines connect the dots of the mean AUPRC scores for two sets of experiments: random sub-selection of genomes (full lines) and sub-selection to keep maximum diversity among the selected genomes (dashed lines). Colour denotes the number of genomes used in the phylogenetic profiles.

In theory, an increase in the number of functional annotations should always be beneficial for phylogenetic profiling: the more training data we have, the better our functional annotation. We were interested in evaluating what effect changing the amount of annotations has on predictive accuracy: we systematically reduced the number of annotations available to annotate our phylogenetic profiles and then evaluated predictive accuracy (Fig. 2).

For the overall predictive accuracy of the 1093 GO terms included in this analysis, removing annotations drastically reduced predictive accuracy. This held across different number of genomes considered in the phylogenetic profiles, and for different genome selection criteria (factors which we discuss in detail below). In particular, upon discarding annotations down to 20% of the current levels, phylogenetic profiling became completely ineffective regardless of the number of species considered (Fig. 2).

At the right end of the curves in Fig. 2, the improvement in predictive accuracy of phylogenetic profiling tapers off beyond 80% of added annotations. However, because we only consider terms that are associated with at least 50 phylogenetic profiles (see Methods), the 1093 GO terms included in this analysis are inherently skewed toward well-annotated terms. Consequently, at the point when 80% of annotations are considered, there often remains enough annotations for terms to be reliably assigned to phylogenetic profiles. Indeed, if we consider an even better annotated subset of 777 GO terms associated with at least 100 profiles each, the tapering beyond 80% becomes more pronounced (Figure A in S1 File).

### How many genomes are enough?

As previous studies have highlighted, including more genomes in phylogenetic profiles is not necessarily beneficial in terms of predictive accuracy [3–5]. Additional genomes in the profiles can influence predictive accuracy in two ways: 1) they may provide useful information for the annotation models, thereby increasing predictive accuracy; 2) they may provide redundant information, which would be at best neutral, or disruptive to methods that do not properly account for the correlation arising from evolutionary relationships between species [14].

**Figure 3:**
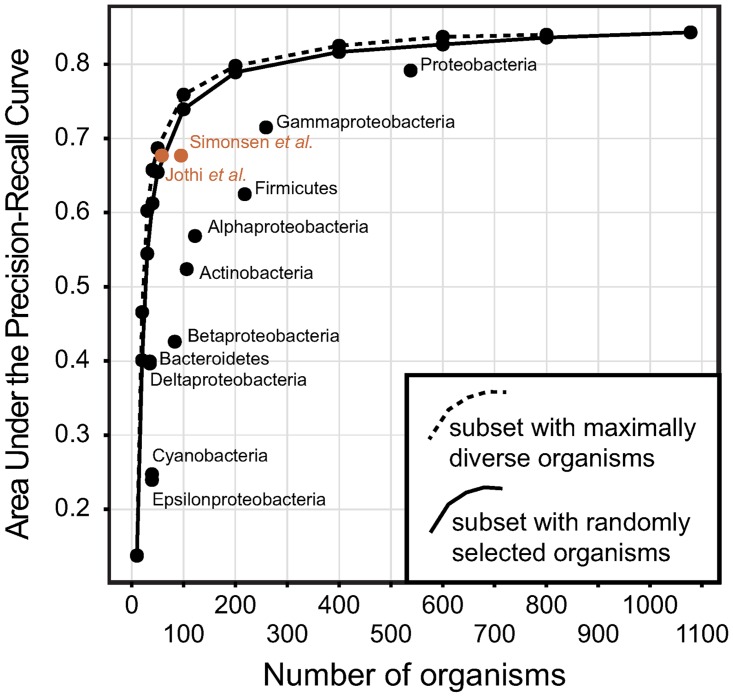
Predictive accuracy of phylogenetic profiling when we change the number of included genomes. Dashed and full lines connect the dots representing the mean AUPRC scores for two sets of experiments: random sub-selection of genomes (full lines) and sub-selection to keep maximum diversity among the selected genomes (dashed lines). Each dot represents the mean AUPRC for the GO terms we use in annotating. The rightmost point denotes the mean AUPRC score when we include all the available bacteria in the OMA 2012 release (1078 bacteria). Separate dots denote the mean AUPRC for subsets of genomes denoted with the label.

To investigate how the number of genomes influences predictive accuracy, we selected genomes to include in phylogenetic profiles either randomly or to obtain maximum phylogenetic diversity (Fig. 3). For each experiment in this and the next section, regardless of the number of genomes used, we kept the annotations of phylogenetic profiles the same: the annotations were those assigned to the phylogenetic profile when we used all the available data, so any difference in predictive accuracy is a consequence of the different genomes used.

The usefulness of adding more genomes is visible in our experiments: the more genomes get included in phylogenetic profiles, the better the predictive accuracy. In fact, contrary to previous reports [5], we obtained best predictive accuracy when we use *all* the available bacteria in our phylogenetic profiles.

It is the effect of adding redundant genomes (i.e., phylogenetically close genomes) that is discussed in many reports in the literature, arguing that for good predictive accuracy a well chosen subset of genomes is imperative [2]. In our experiments, redundant information in phylogenetic profiles did not lead to accuracy degradation because our algorithm properly deals with potential correlation among profiles (see Methods). As a result, additional genomes are never detrimental in terms of predictive accuracy.

However, additional genomes in the phylogenetic profiles result in a heavier computational burden. This raises the question of how many genomes are required for phylogenetic pattern methods to be accurate. Our analyses suggest that independent of the amount of annotations considered, phylogenetic profiling only marginally improves beyond ∼100 genomes, with no practical difference beyond ∼400 genomes (Fig. 3).

### Influence of genome selection on phylogenetic profiling: maximal phylogenetic diversity performs best and can nearly be achieved through random sampling

A reasonable strategy for the selection of genome subsets is to keep the largest phylogenetic diversity within the subset, either manually selecting the subset [4] or using a computational method [5]. In fact, preserving the maximum diversity within a set of genomes used for phylogenetic profiling was the best strategy in our experiments (Fig. 3).

To get a better insight into the influence of larger phylogenetic diversity on the predictive accuracy of phylogenetic profiling, we focused on two possible extremes when selecting our subsets: 1) we included phylogenetically close genomes that form a clade; 2) we used two phylogenetically diverse previously published sets of genomes shown to perform well in phylogenetic profiling.

First, we focused on five major phylogenetic groups of sequenced bacteria: Proteobacteria (538 genomes), Firmicutes (218 genomes), Actinobacteria (106 genomes), Bacteroidetes (35 genomes), and Cyanobacteria (39 genomes). We carried out three groups of experiments. First, we created phylogenetic profiles using only genomes in the respective groups. Second, we created phylogenetic profiles that include a random selection of bacteria such that the number of randomly selected bacteria is the same as the number of bacteria in each major group. We performed ten random selections of genomes, and the results reported in this work—the AUPRC values—are the mean values of these experiments. Third, we created phylogenetic profiles that include a selection of bacteria that maximizes phylogenetic diversity within the set such that the number of selected bacteria is the same as the number of bacteria in each major group.

In our experiments, phylogenetic profiling based only on clades is a poor strategy: the maximally diverse set always outperforms the randomly chosen set (Fig. 3); even the dataset based on our largest clade of bacteria, Proteobacteria (538 genomes), is outperformed by the dataset containing the same number of maximally diverse bacteria (Fig. 3 and Figure B in S1 File).

Second, we investigated how well our phylogenetic profiling method works with published subsets of genomes: we used two subsets available in the literature: a manually selected subset by Jothi *et al*. [4] and a computationally selected subset by Simonsen *et al*. [5].

Jothi *et al*.[4] evaluated a number of manually curated sets of genomes and found that a non-redundant set containing members from all three kingdoms dubbed BAE3a had on average the best accuracy when predicting functional linkages. In our experiments, the predictive accuracy using the BAE3a set is inferior to the predictive accuracy when using the same number of genomes, but selected from our set of 1078 bacteria, be it a random selection of 58 genomes, or a selection to obtain a maximally diverse set of 58 genomes (Figure C in S1 File). Our selection was made from a much more diverse set of genomes than the set available in 2007 when Jothi *et al*. made their selection, and the difference in predictive accuracy might indicate the advantage of having a larger pool of sequenced genomes to chose from.

More recently, Simonsen *et al*. [5] presented methods to automate the selection of genome subsets used in phylogenetic profiling; they focused on the inference of protein-protein interactions. Similar to our results when predicting function, they showed that genome selection with the largest phylogenetic diversity improves predictive accuracy. By removing closely related genomes from the set, they created a reference genome set that improved the predictive accuracy compared to both their full set of 980 genomes and the BAE3a set of Jothi *et al*. [4]. We reconstructed this set and compared its predictive accuracy in our framework (Fig. 3). In our experiments, the Simonsen *et al*. [5] dataset has a similar predictive accuracy as the BAE3a dataset and shows slightly lower, but similar predictive accuracy as our datasets obtained by either random sampling or a sampling that preserves the highest diversity within the dataset (Fig. 3 and Figure C in S1 File).

### Our main conclusions hold under the Open World Assumption

One challenge in assessing function prediction is to account for the inherent incompleteness of biological databases. Indeed, because gene products can have multiple functions, absence of functional annotation is not evidence of absence of function—a notion referred to as the Open World assumption (OWA) [17, 18]. In this section, we provide corroborating evidence that our findings hold in light of the OWA.

First, we ask: how are our analyses affected by the OWA? The predictive accuracy metric we used in this study (*Area Under the Precision-Recall Curve*, AUPRC) is computed from the out-of-bag error: for each iteration of the experiment, a predictor is built using ∼63% of the data selected randomly. To each sample in the remaining ∼37%—samples *not* used to train the predictor—the predictor assigns the probability that a given OMA group of gene products is associated with each GO term. Thus, comparing these predictions with the actual GO term(s) assigned to each gene product yields True Positives, False Positives, True Negative, and False Negative counts, from which Precision and Recall can be computed. The AUPRC measures the area under the curve denoted by the values of Recall (x-axis) and Precision (y-axis).

However, counting predictions that are absent from the reference set as False Positives yields biased results under the OWA: absence from the reference set could be due to mere incompleteness of the database.

To control for the effect of this bias on our conclusions, we performed two sets of control experiments: 1) we repeated our analyses on a dataset that is likely to suffer less bias from violating the OWA: gene products with at least five annotations in the UniProt-GOA database. All else being equal, by virtue of having more annotations, these gene products can be expected to be more comprehensively annotated. 2) We repeated our analyses on a dataset that is likely to suffer more bias from violating the OWA than our normal dataset: we randomly left out 60% of all the annotations in the UniProt-GOA database.

**Figure 4:**
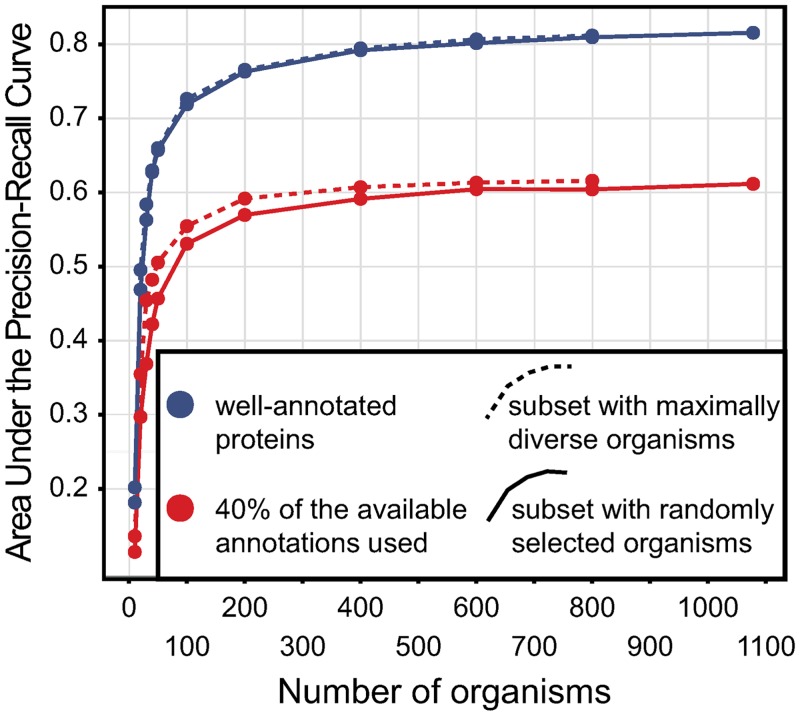
Predictive accuracy of phylogenetic profiling when we control for the influence of the Open World Assumption. Two sets of experiments are denoted with colours: experiments when we include only the well-annotated proteins (purple) and experiments where we randomly remove 60% of the available annotations (red). Dashed and full lines connect the dots of the mean AUPRC scores for two sets of experiments: random sub-selection of genomes (full lines) and sub-selection to keep maximum diversity among the selected genomes (dashed lines). Each dot represents the mean AUPRC for the GO terms we use in annotating. The final point denotes the mean AUPRC score when we include all the available bacteria in the used OMA database release (1078 bacteria).

Both alternate datasets yielded highly consistent results (Figs. 3 and 4). When we included in our phylogenetic profiles only the well-annotated proteins, we replicated the shape of the curve in Fig. 3: selection to obtain maximally diverse sets of genomes outperforms the random selection, but marginally so; the effect of adding more than ∼100 genomes diminishes. Similarly, when we excluded 60% of annotations, the overall predictive accuracy reduced, but the shape of the curve followed the shape of the curve in Fig. 3, starting to saturate around ∼100 genomes. Thus, this indicates that our main findings are not affected by a violation of the OWA.

### Conclusion

When phylogenetic profiling was first described fifteen years ago [1], even with the few sequenced genomes that were available back then, the method provided new insights into protein function of *Escherichia coli*. Since then, there have been numerous refinements of the method, many focusing on the optimal selection of genomes for increasing the predictive accuracy of functional annotation.

Here, we have shown that an increase in available input data alone has drastically improved the predictive accuracy of phylogenetic profiling. However, adding more genomes beyond ∼100 only has a marginal effect on predictive accuracy. Furthermore, we have confirmed that maximising phylogenetic diversity yields best results, and that a straightforward random selection procedure from a broad range of species nearly achieves the same result.

Should the future releases of the annotation databases provide more of the more specific functional annotations, phylogenetic profiling will benefit and propagate these annotations to non-model organisms. Moreover, with the increase in the number of sequenced genomes, we will have a larger set of genomes to choose from and thereby further improve the phylogenetic profiles.

Phylogenetic profiling was a good idea fifteen years ago; owing to the additional functional and genomic data we now have available, it is an even better one today.

## Methods

### Phylogenetic profiling

To explore the extent to which newly sequenced genomes and the new information in the annotation databases influence phylogenetic profiling, we focused on a recently published method for phylogenetic profiling [7]. In our implementation, we tracked the patterns of presence or absence of homologs inferred by the OMA algorithm [19]; we included both orthologs and paralogs because our previous research showed that paralogs, when used in addition to orthologs in phylogenetic profiling, improve predictive accuracy [20]. Therefore, each row in the binary phylogenetic profile represents one OMA group, and a 1 or a 0 represents the presence or absence of an OMA group member and its orthologs or paralogs in the included genomes.

We assigned Gene Ontology (GO) terms [15, 21] to OMA phylogenetic profiles: when at least half of the OMA group members—those homologs that are all mutually orthologous—are annotated with the respective GO term, we assign the GO term to the OMA group itself, and consequently also to the OMA phylogenetic profile. For the functional annotations, we used the releases of the UniProt-GOA database available from the UniProt-GOA FTP site: for each denoted year, we used the first yearly release. If not otherwise noted, we only included GO terms that are assigned to at least 50 OMA phylogenetic profiles.

To annotate the phylogenetic profiles, we included GO terms assigned using evidence codes EXP, IMP, IGI, IPI, IEP, and IDA, as well as selected GO annotations assigned using the evidence code IEA: those inferred from UniProtKB keywords, UniProt Subcellular Location terms, Enzyme Commission numbers, and InterPro. In a recent report, we showed these electronic annotations are highly reliable, particularly in the prokaryote *Escherichia coli* [20]. To obtain functional annotations, we used various releases of the UniProt-GOA database, all of them freely available at the UniProt-GOA FTP site.

We presented this phylogenetic profile to a machine learning algorithm based on decision trees [22], thereby obtaining a model for functional annotation, as well as the estimation of predictive accuracy for each GO term. More details on the algorithm and our implementation of phylogenetic profiling are available in the original publication [7].

### Genome data

For the patterns of presence or absence, we used the data available in the December 2012 release of the OMA database. This release contained 1078 Bacteria, 107 Archaea, and 135 Eukaryotes. Each of the subsets of genomes in our work is derived from this set of genomes.

In this work, we focused on Bacteria because this is by far the most numerous kingdom in the OMA database. Moreover, eukaryotic phylogenetic profiles showed limited success, both in the literature [4, 23–25], as well as in our own experiments (Figure D in S1 File). Interestingly, Jothi *et al*. showed best average predictive accuracy for the dataset containing a few selected eukaryotes, while the predictive accuracy deteriorated when their datasets included all the eukaryotes available at the time: they note that the over-representation of parasitic eukaryotes and vertebrates made eukaryotes less useful for phylogenetic profiling.

### Species selection

We considered four strategies to select genome subsets: random selection, selection to preserve the maximum diversity among genomes, selecting genomes that belong to a particular clade, and previous strategies from the literature.

When selecting for maximum diversity, we iterated through the distance matrix used to construct the species phylogenetic tree in the OMA database: we searched for the two closest species in the matrix and excluded the one with fewer GO annotations, repeating this process until we were left with the desired number of genomes. For these experiments that evaluate the influence of the number and diversity of genomes on phylogenetic profiling, we only changed the number of genomes in phylogenetic profiles, keeping as the annotations of phylogenetic profiles those assigned when we used all the available data.

We evaluated the predictive accuracy of our implementation of phylogenetic profiling on two published subsets of genomes. First, a manually curated subset of genomes by Jothi *et al*. [4]: we created a subset of strains that closely matches that named BAE3a, for which they report best overall predictive accuracy. The December 2012 OMA database release does not contain three strains of *Chlamydophila pneumoniae*: AR39, CWL029, and J138. They were replaced by the strain available in the OMA database: LPCoLN. In addition, the OMA database does not contain three of the strains listed in the BAE3a set; we replaced those with closely related strains. The final dataset had 58 genomes: their selection very closely resembled the BAE3a set.

The second published set was derived from Simonsen *et al*. [5]: Martin Simonsen kindly provided the list of 100 genomes that showed best predictive accuracy for *E. coli* phylogenetic profiling. This set had been automatically obtained using their method of Tree-Based Search. Out of these 100, we did not have 4 strains in the OMA database: *Xenorhabdus nematophila* (strain ATCC 19061 / DSM 3370 / LMG 1036 / NCIB 9965 / AN6), *Kangiella koreensis* (strain DSM 16069 / KCTC 12182 / SW-125), *Escherichia fergusonii* (strain ATCC 35469 / DSM 13698 / CDC 0568–73), and *Tolumonas auensis* (strain DSM 9187 / TA4). We omitted these, thus obtaining a dataset with 96 genomes.

### Evaluating predictive accuracy

To infer our functional annotation models, we used the implementation of the Random Forest algorithm [26] in the CLUS software [27]. This software is a Predictive Clustering System that builds decision trees able to deal with multiple hierarchically organized class labels, such as terms from the Gene Ontology, for Hierarchical Multi-label Classification (HMC). The Random Forest Ensemble approach used—combining multiple decision trees—obtains better predictive accuracy than could be obtained from individual decision trees [22].

**Figure 5:**
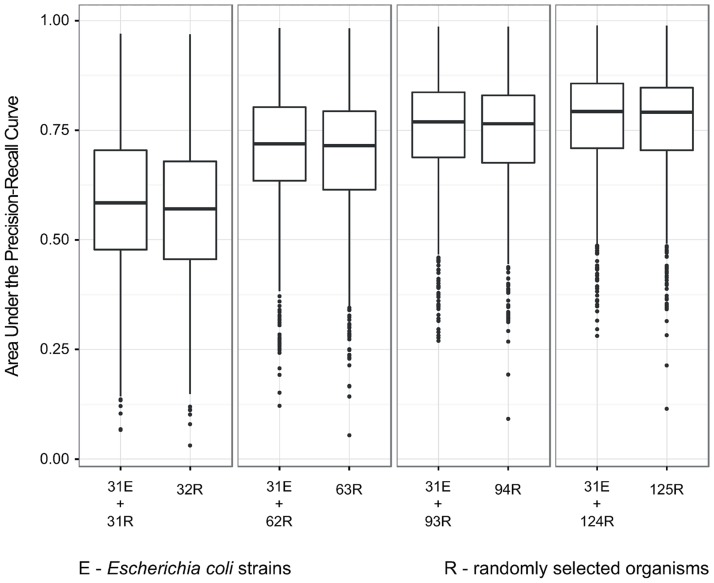
Predictive accuracy of phylogenetic profiling was not affected when we used many strains of the same organism. We used 31 strains of *Escherichia coli* and we added to this set: A) 31 random organisms, B) 62 random organisms, C) 93 random organisms, and D) 124 random organisms. Each plot in a panel corresponds either to the combination of the 31 *E. coli* strains and the randomly selected organisms (left) or just the randomly selected organisms (right). Each boxplot summarizes AUPRC scores for GO terms in the dataset indicated on the x-axis. Lower, mid, and upper horizontal lines denote the first quartile, median and the third quartile, respectively; vertical lines reach 1.5 interquartile range from the respective quartile or the extreme value, whichever is closer. Each plot summarizes the results for ten independent random organism selections.

The first important feature of the Random Forest Ensemble approach is that individual decision trees are constructed by searching over a random *subset* of the available decisions—in our case presence/absence patterns of OMA members in genomes—when splitting a node. These individual trees are combined to produce the final result. Because of this combination, Random Forest reduces the influence of redundant decision points, patterns of presence or absence, that would influence methods relying on distance metrics [3]. Consequently, when testing whether adding new genomes is beneficial for phylogenetic profiling, the addition of possibly redundant decision points (e.g., phylogenetically close genomes) will not have a detrimental effect, while the addition of possibly useful decision points (e.g., phylogenetically distant genomes) will increase predictive accuracy (Fig. 5).

The second important feature of the Random Forest Ensemble approach is that each decision tree is constructed from a different sample of the training dataset: the bagging (Bootstrap aggregating) methodology samples with replacement [28]. In this way, about 63% of unique samples are selected for training the decision tree and produce the functional annotation model—the decision tree that can infer GO terms assigned to an OMA group based on the presence/absence of OMA members in the genomes.

The procedure above allowed us to calculate the True Positives, False Positives, and False Negatives, thereby obtaining the corresponding AUPRC score. For each experiment, we repeated the procedure 500 times, averaging the probabilities of this ensemble of decision trees, thereby obtaining the final estimates of Precision, Recall, and the AUPRC.

### Analysed data

The file that contains the AUPRC values obtained for each GO term in each of the experiments performed for this work can be downloaded from http://lab.dessimoz.org/14_phylprof. On the same web page, we also provide a list of all the 1093 GO terms that formed the backbone of our analysis. The CLUS algorithm we used in our work is freely available from http://dtai.cs.kuleuven.be/clus/ and the OMA database can be accessed at http://omabrowser.org (we used the December 2012 release).

## Supporting Information

S1 Data AUPRC values obtained for each GO term in each our experiments. (ZIP)

S2 Data List of GO terms used in reduced set (777 terms). (ZIP)

S3 Data List of GO terms used in main analyses (1093 terms). (ZIP)

S4 Data List of organisms used for the BAE3a dataset. (ZIP)

S1 File Supporting figures. (PDF)
