# Supplementary material for: Phylogenetic Profiling: How Much Input Data Is Enough?
Source: PLoS One. 2015 Feb 13;10(2):e0114701. doi: 10.1371/journal.pone.0114701 (PMC4332489; doi:10.1371/journal.pone.0114701)
Supplement: S1 File — (PDF) [file pone.0114701.s005.pdf]

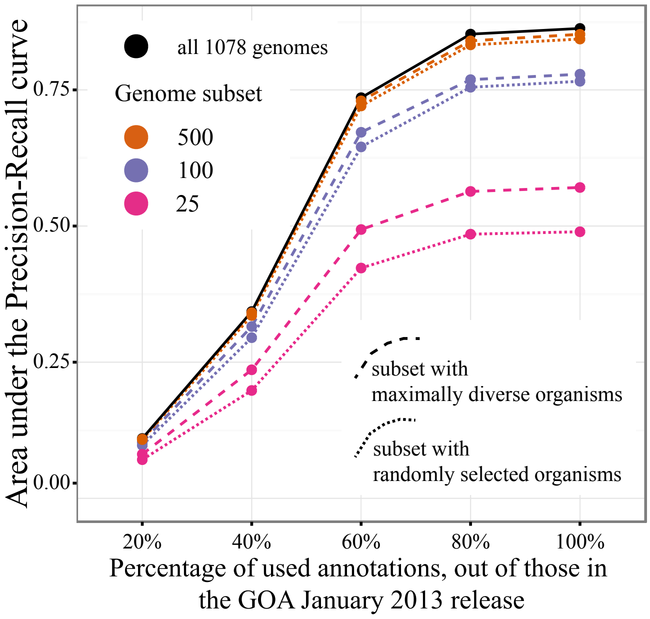

Figure A. Performance of phylogenetic profiling, measured in AUPRC, when we reduce the number of annotations used for phylogenetic profiling. For each of the experiments denoted on the x-axis, we only used a fraction of the available annotations in the most recent dataset. Dashed and full lines connect the dots of the mean AUPRC scores for two sets of experiments: random sub-selection of genomes (full lines) and sub-selection to keep maximum diversity among the selected genomes (dashed lines). Colour denotes the number of genomes used in the phylogenetic profiles. For these experiments, we evaluated predictive accuracy for the 777 GO terms that were assigned to at least 100 phylogenetic profiles.

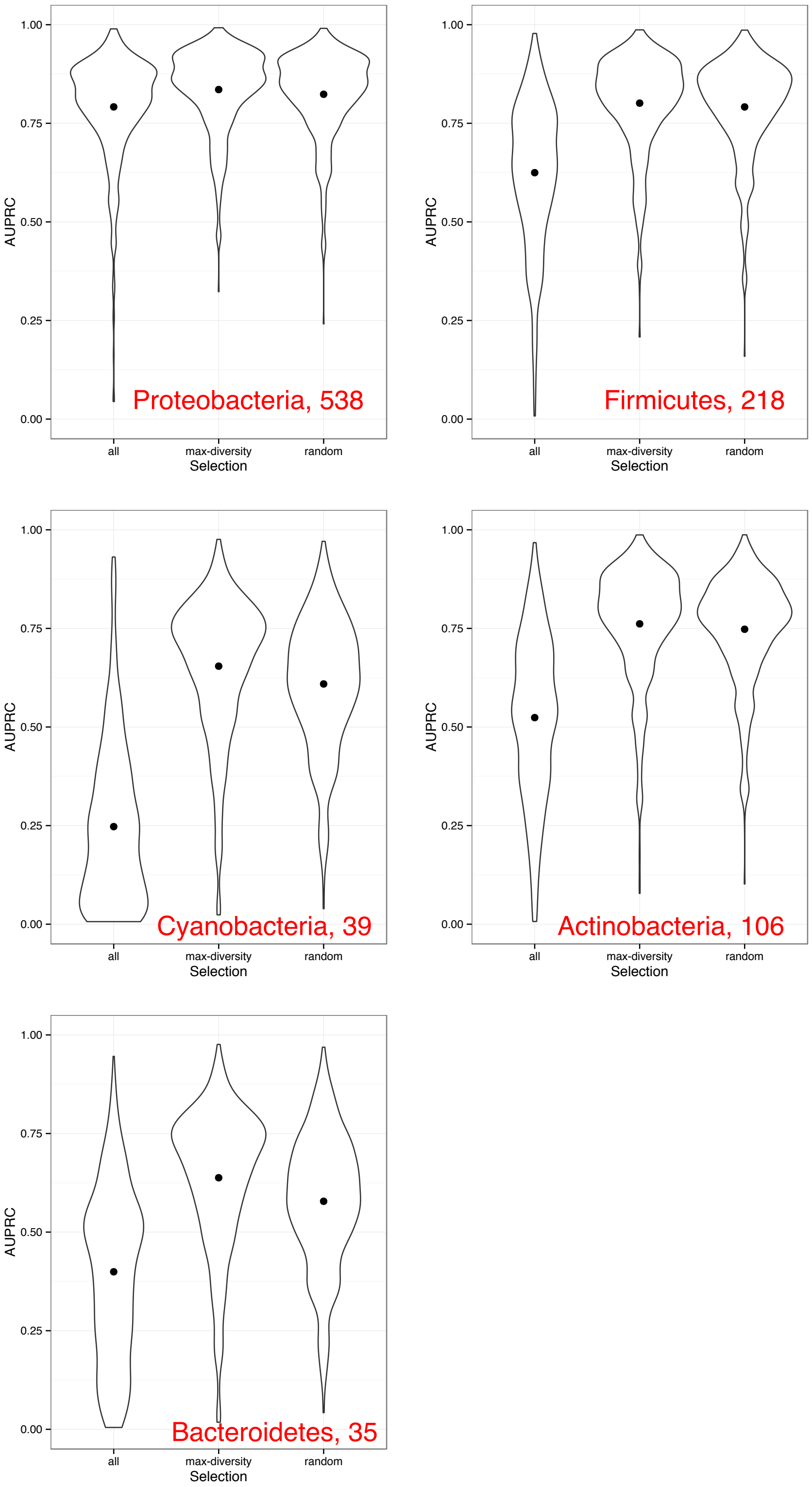

Figure B. Performance of phylogenetic profiling, measured in AUPRC, on five major groups of sequenced bacteria, available in the OMA database Dec 2012 release. A) Proteobacteria (538 organisms), B) Firmicutes (218 organisms), C) Actinobacteria (106 organisms), D) Bacteroidetes (35 organisms), and E) Cyanobacteria (39 organisms). Each plot in a panel corresponds either to the group of bacteria (left), a subset of organisms with the maximum phylogenetic diversity, having the same number as the number of organisms in the group of bacteria (middle), or a random

subset of organisms, having the same number as the number of organisms in the group of bacteria (right). The area of each violin plot summarizes the distribution of GO terms according to the AUPRC value: the area of the plot corresponds to the probability density of GO terms at different values of AUPRC. The black dot denotes the mean value of AUPRC for the respective dataset.

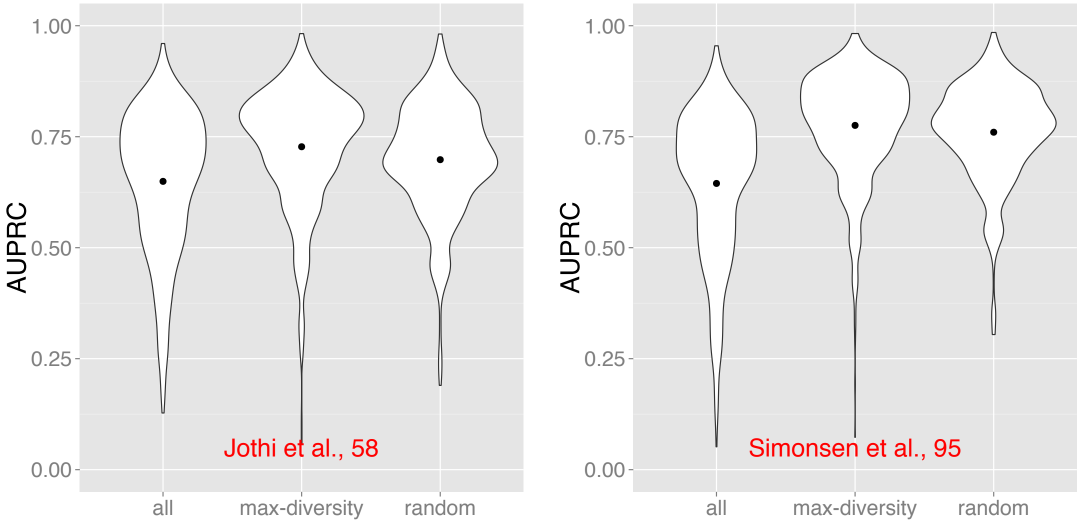

Figure C. Performance of phylogenetic profiling, measured in AUPRC, for two triplets of datasets with the same number of organisms, but of different composition. A) Manually assembled dataset shown to have the best average score among the datasets tested by Jothi *et* al., 2007. For the left panel, the manually selected 58 organisms, predominantly bacteria; for the middle panel, randomly selected 58 bacteria from our pool of 1078 bacteria; for the right panel, 58 bacteria selected to have the highest phylogenetic diversity in the set. B) Automatically selected set, shown to be best among those examined by Simonsen *et al.*, 2012. For the left panel, the 96 automatically selected genomes; for the middle panel, randomly selected 96 bacteria from our pool of 1078 bacteria; for the right panel, 96 bacteria selected to have the highest phylogenetic diversity in the set.

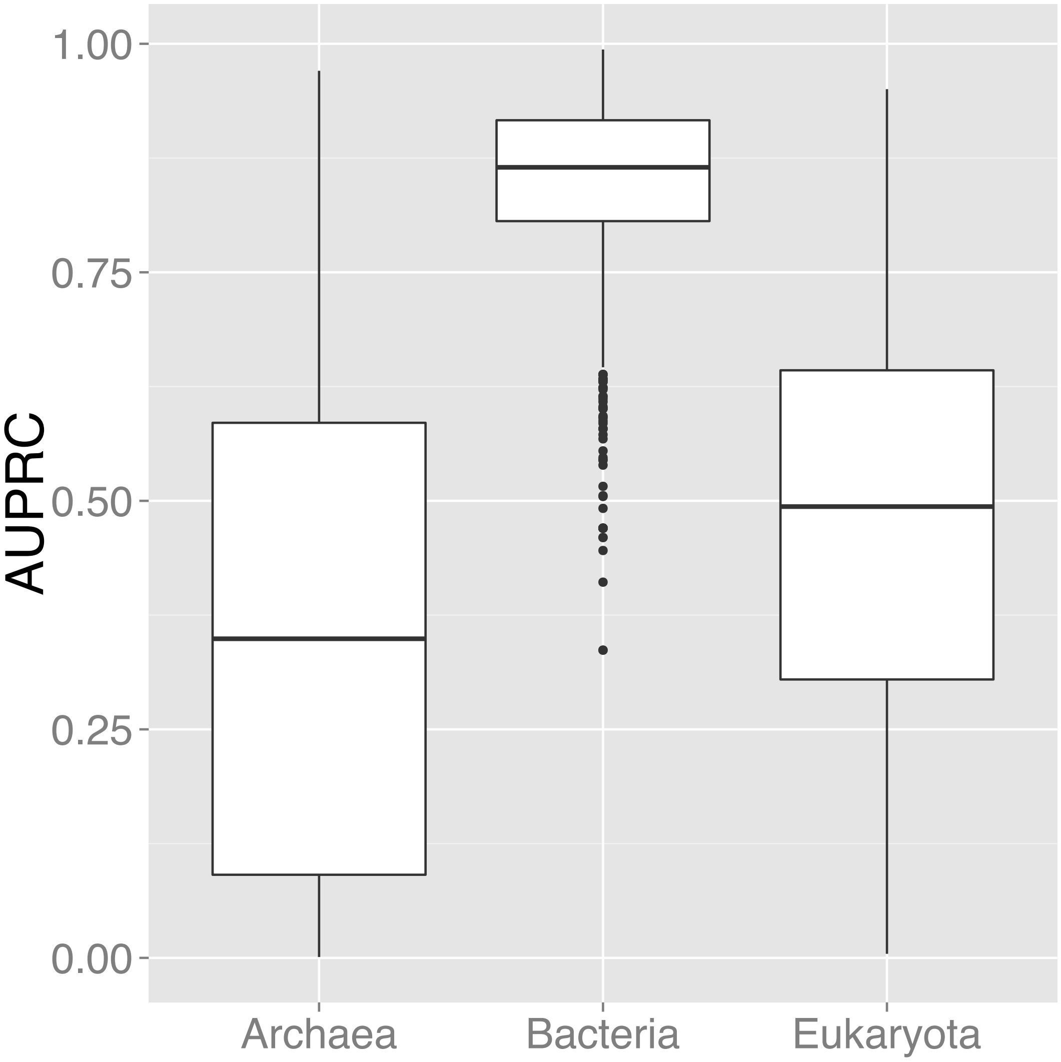

Figure D. Performance of phylogenetic profiling on three different kingdoms: eukaryotes, bacteria, and archaea. Each boxplot summarizes Area Under the Precision-Recall Curve (AUPRC) scores for the dataset indicated on the x-axis. Lower, mid, and upper horizontal lines denote the first quartile, median and the third quartile, respectively; vertical lines reach 1.5 interquartile range from the respective quartile or the extreme value, whichever is closer.
